# Impact of Hyperoxia and Hypocapnia on Neurological Outcomes in Patients with Aneurysmal Subarachnoid Hemorrhage: A Retrospective Study

**DOI:** 10.1155/2019/7584573

**Published:** 2019-12-06

**Authors:** Kin Chio Li, Catherine Wing Yan Tam, Hoi-Ping Shum, Wing Wa Yan

**Affiliations:** ^1^Department of Intensive Care, Pamela Youde Nethersole Eastern Hospital, 3 Lok Man Road, Chai Wan, Hong Kong, China; ^2^Department of Radiology, North District Hospital, 9 Po Kin Road, Sheung Shui, Hong Kong, China

## Abstract

In recent decades, there is increasing evidence suggesting that hyperoxia and hypocapnia are associated with poor outcomes in critically ill patients with cardiac arrest or traumatic brain injury. Yet, the impact of hyperoxia and hypocapnia on neurological outcome in patients with subarachnoid hemorrhage (SAH) has not been well studied. In the present study, we evaluated the impact of hyperoxia and hypocapnia on neurological outcomes in patients with aneurysmal SAH (aSAH). Patients with aSAH who were admitted to the intensive care unit (ICU) of a tertiary hospital in Hong Kong between January 2011 and December 2016 were retrospectively recruited. Patients' demographics, comorbidities, radiological findings, clinical grades of SAH, PO_2,_ and PCO_2_ within 24 hours of ICU admission, and Glasgow Outcome Scale (GOS) at 3 months after admission were recorded. Patients with a GOS score of 3 or less were considered having poor neurological outcomes. Among the 244 patients with aSAH, 122 of them (50%) had poor neurological outcomes at 3 months. Early hyperoxia (PO_2_ > 200 mmHg) and hypercapnia (PCO_2_ > 45 mmHg) were more common among patients with poor neurological outcomes. Logistic regression analysis indicated that hyperoxia independently predicted poor neurological outcomes (OR 3.788, 95% CI 1.131–12.690, *P*=0.031). Classification tree analysis revealed that hypocapnia was associated with poor neurological outcomes in patients who were less critically ill (APACHE < 50) and without concomitant intracranial hemorrhage (ICH) or intraventricular hemorrhage (IVH) (adjusted *P*=0.006, *χ*^2^ = 7.452). These findings suggested that hyperoxia and hypocapnia may be associated with poor neurological outcomes in patients with aSAH.

## 1. Introduction

Despite accounting only 3–5% of all causes of stroke, aneurysmal subarachnoid hemorrhage (aSAH) remains a significant cause of stroke-related mortality, with local data showing a 1-year mortality of 19% [1]. Another distinct feature of aSAH patients is that they are typically younger than those having ischemic stroke or intracranial hemorrhage, with the peak occurrence of aSAH between the ages of 50 and 60 [2]. Together with the functional and cognitive deficits among survivors, SAH imposes a heavy burden to healthcare comparable with other forms of stroke [3].

Abnormal PO_2_ and PCO_2_ are not uncommon among neurosurgical patients in the intensive care unit (ICU). In recent decades, there is an increasing concern of the effects of hyperoxia and hypocapnia on outcomes in patients who have sustained neurological insults. Several clinical studies involving cardiac arrest survivors and patients with traumatic brain injury or cerebrovascular accident suggested that hyperoxia and hypocapnia may be associated with poor outcomes [4–6]. Current guidelines do not specify the optimal PO_2_ and PCO_2_ level for patients with aSAH [7], while several retrospective studies yielded contradicting results. The present study aimed to evaluate the impact of hyperoxia and hypocapnia on neurological outcomes in patients with aSAH.

## 2. Materials and Methods

### 2.1. Study Design and Population

This is a single center, retrospective cohort study performed at a tertiary hospital in Hong Kong. Patients with aSAH who were admitted to the adult ICU between January 1, 2011, to December 31, 2016, were retrospectively recruited. For patients with a history of recurrent ICU admission, only data from their first ICU admission were included. Patients with traumatic SAH (tSAH) and those with missing data were excluded. The study protocol and procedures were approved by the Hong Kong East Cluster Ethics Committee. Written consent was waived due to the retrospective, anonymous nature of the study.

### 2.2. Data Collection and Outcome Measures

Written clinical notes and electronic patient records were reviewed. Information on patients' demographics and comorbidities, clinical findings in terms of World Federation of Neurosurgical Society (WFNS) grades, radiological findings including location and size of aneurysms, presence of intracranial hemorrhage (ICH), or intraventricular hemorrhage (IVH), and Modified Fisher radiological grading scale were extracted. The presence of cerebral vasospasm was assessed by transcranial doppler (TCD). The degree of vasospasm was classified into three groups based on the mean flow velocities: mild (>100–130 cm/s), moderate (>130–200 cm/s), and severe (>200 cm/s). A nurse specialist performed all the scans. For cases with difficulties in assessment with TCD, computer tomography angiography or digital subtraction angiography were used.

Arterial blood gas results during initial 24 hours of ICU admission were reviewed. We defined hyperoxia as having at least one blood gas sample with PO_2_ >200 mmHg during this period. Hypocapnia was defined as having at least one blood gas sample with PCO_2_ <30 mmHg, while a PCO_2_ result of >45 mmHg was considered to be hypercapnia. For patients who underwent brainstem death tests, their blood gas results obtained within a 12 hour-period before the tests were excluded.

The primary outcome was the Glasgow Outcome Scale (GOS) score at 3 months after ICU admission. A GOS score of 3 or less represented a poor neurological outcome, whereas the GOS score of 4 or 5 indicated a good outcome. Other outcome data included length of stay (LOS) in the ICU and hospital, as well as mortality.

### 2.3. Statistical Analysis

An epidemiological study by Wong et al. found that the local incidence of SAH was 7.5 per 100,000 person-years [1]. The calculated sample size for our study was 94 with 95% confidence interval and 10% margin of error to provide representative findings.

Recruited patients were divided into two groups according to their neurological outcomes, i.e., GOS scores at 3 months. Demographics and other baseline characteristics were compared with Student's *t* test or Mann–Whitney *U* test for continuous variables, and Pearson Chi-squared test or Fisher's exact test for categorical variables where appropriate. Results were expressed as means and standard deviations (SDs), or as number of cases and percentages.

Logistic regression analysis was used to assess the independent predictors of poor neurological outcomes. Continuous variables were converted to categorical variables for ease of statistical analyses. Cutoff values were determined based on receiver operating characteristic (ROC) curve analysis. Predictor variables with *P* < 0.1 in univariate analyses were included in the regression model, excluding those with collinearity. They included age >55 years old, APACHE IV score >50, gender, WFNS grade >3, Modified Fisher Grading Scale >2, presence of ICH/IVH, aneurysm within posterior circulation, decision for endovascular intervention, hyperoxia (PO_2_ > 200 mmHg), and hypercapnia (PCO_2_ > 45 mmHg). The fit of regression model was assessed using the Hosmer and Lemeshow test, Cox and Snell R Square test, and Nagelkerke R square test.

Classification tree analysis was employed to identify predictors and determine their relationship with poor neurological outcomes. This is a standard data analytical approach for identifying mutually exclusive and exhaustive subgroups within a population with shared certain characteristics that influence the dependent variable, i.e., neurological outcome. Chi-squared tests were performed repeatedly, and a new node was created if the result was significant and maximized differences in the dependent variable. Predictor variables with the smallest Bonferroni-adjusted *P* value and giving the most significant split were chosen. A terminal node was produced when the smallest adjusted *P* value for any predictor was not significant, or when there were less than 50 cases in the child node.

Statistical analyses were conducted using the Statistical Package for the Social Sciences for Windows version 20 (SPSS Inc., Chicago II, USA).

## 3. Results and Discussion

### 3.1. Results

#### 3.1.1. All Patients

During the study period, 244 patients with aSAH were recruited (Table 1). The mean age of patients was 57.7 years, and 36.4% of patients were male. The mean lowest Glasgow Coma Scale (GCS) on day 1 of ICU admission was 8. 43.9% of patients had WFNS grades of 4 or 5 (16.4% and 27.5%, respectively), and 75% had Modified Fisher grades of 3 or 4 (22.1% and 52.9%, respectively). 63.5% of patients had concomitant ICH or IVH. Aneurysms were more commonly found in anterior circulation (62.7%); 27% were located in posterior circulation. Aneurysms' location was not identified in 25 cases (10.3%). Of all cases, 55.7% received interventional radiological procedures, whereas 21.7% of cases underwent surgical clipping. External ventricular drainage was performed in 53.7% of cases. Hyperoxia (PO_2_ > 200 mmHg) was noted in 16% of patients. 33.2% of patients experienced hypocapnia (PCO_2_ < 30 mmHg) while hypercapnia (PCO_2_ > 45 mmHg) was noted in 24.6% of cases.

#### 3.1.2. Comparison between Patients with Poor and Favorable Outcomes

**Figure 1:**
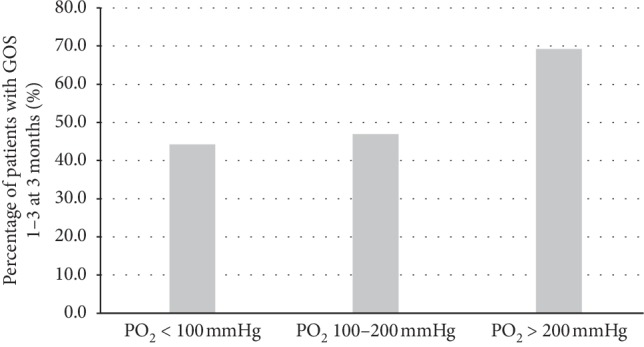
PO_2_ level within first 24 hours of ICU admission in patients with poor neurological outcomes. *P* < 0.031 for trend analysis. Abbreviations: ICU = intensive care unit; GOS = Glasgow Outcome Scale.

Of all the recruited cases, 50% (122) had poor neurological outcomes (Table 1). Compared to patients with favorable neurological outcomes, they significantly differed in terms of age (*P* < 0.001), sex (*P*=0.024), APACHE IV score (*P* < 0.001), APACHE IV risk of death (*P* < 0.001), GCS on the first day of admission (*P* < 0.001), WFNS grading (*P* < 0.001), Modified Fisher radiological grading (*P* < 0.001), location of aneurysm (*P*=0.002), presence of concomitant ICH/IVH (*P* < 0.001), endovascular intervention (*P* < 0.001), external ventricular drainage (*P* < 0.001), length of stay in the ICU (*P*=0.036), length of hospital stay (*P*=0.009), and mortality (*P* < 0.001). There was no statistically significant difference in the number of patients with vasospasm between the two groups (*P*=0.153). Of our particular interest, hyperoxia (*P*=0.009) and hypercapnia (*P* < 0.001) were more commonly seen among patients with GOS scores of 3 or less within first 24 hours of ICU admission. There was no statistically significant difference in the number of patients with hypocapnia between the two groups (*P*=0.683). Patients with poor neurological outcomes had statistically higher maximum PO_2_ level (161.4 ± 76.1 mmHg vs. 141 ± 43.4 mmHg, *P*=0.011), maximum PCO_2_ (44.9 ± 14.4 mmHg vs. 39.0 ± 6.3 mmHg, *P* < 0.001), and average PCO_2_ level (38.7 ± 8.8 mmHg vs 35.6 ± 4.6 mmHg, *P*=0.001). Among patients with poor neurological outcomes (Figure 1), 69.2% of cases once had hyperoxia (PO_2_ > 200 mmHg) within first 24 hours of ICU admission, which is significantly higher than those with PO_2_ levels between 100–200 mmHg (46.9%) and <100 mmHg (44.2%) (*P* < 0.031 for trend analysis).

#### 3.1.3. Independent Predictors of Poor Neurological Outcome

Logistic regression analysis (Table 2) indicated that hyperoxia (OR 3.788, 95% CI 1.131–12.690, *P*=0.031), APACHE IV score >50 (OR 5.403, 95% CI 2.456–11.886, *P* < 0.001), age >55 (OR 4.065, 95% CI 1.857–8.898, *P* < 0.001), presence of ICH/IVH (OR 3.644, 95% CI 1.602–8.292, *P*=0.002), aneurysms involving posterior circulation (OR 3.685, 95% CI 1.477–9.193, *P*=0.005), Modified Fisher Grading Scale > 2 (OR 3.539, 95% CI 1.465–8.550, *P*=0.005), and WFNS grades >3 (OR 2.937, 95% CI 1.270–6.794, *P*=0.012) independently predicted poor neurological outcomes. On the contrary, patients who had underwent radiological intervention was shown to have better outcomes (OR 0.382, 95% CI 0.170–0.859, *P*=0.020).

#### 3.1.4. Classification Tree Analysis

**Figure 2:**
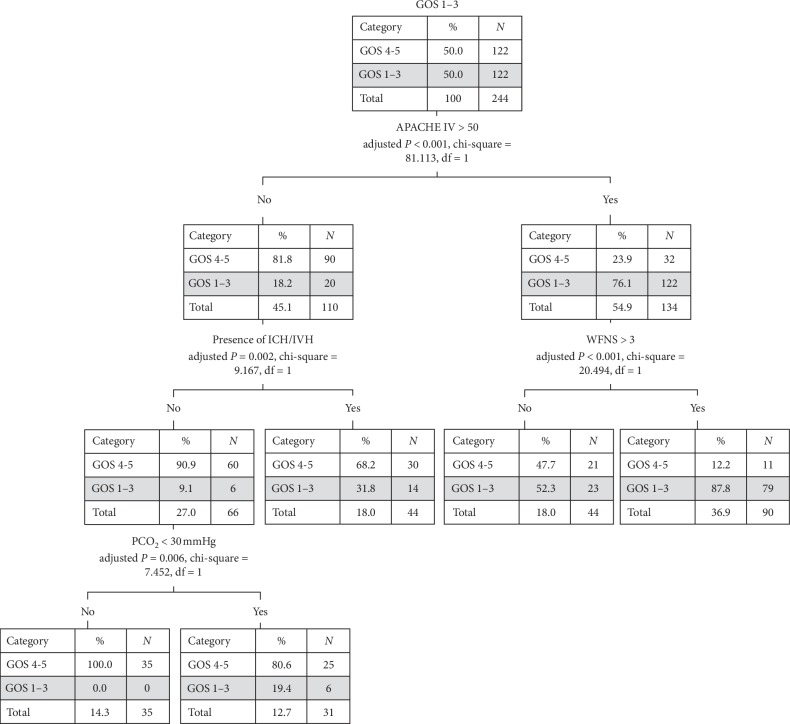
Classification tree model for determining factors of poor neurological outcome. Abbreviations: GOS = Glasgow Outcome Scale; APACHE = Acute Physiology and Chronic Health Evaluation; ICH = intracranial hemorrhage; IVH = intraventricular hemorrhage; WFNS = World Federation of Neurosurgical Society; *N* = number; df = degrees of freedom.

The classification tree model in Figure 2 outlined the predicting factors of poor neurological outcomes. For patients with less severe illness, i.e., APACHE IV score <50, presence of concomitant ICH/IVH-predicted neurological outcome (adjusted *P*=0.002, *χ*^2^ = 9.167). In patients with no ICH/IVH, hypocapnia became the predictor of poor neurological outcomes (adjusted *P*=0.006, *χ*^2^ = 7.452). Among the 31 patients with hypocapnia, 6 (19.4%) had poor neurological outcomes, while there was none in the other group without hypocapnia. Among those with more severe illnesses (APACHE IV score ≥ 50), their neurological outcomes were affected by the WFNS grading score (adjusted *P* < 0.001, *χ*^2^ = 20.494).

### 3.2. Discussion

While some preclinical studies have suggested the beneficial effects of normobaric oxygen in experimental animal models of focal cerebral ischemia [8], there is mounting evidence suggesting that liberal use of supplemental oxygen in critically ill patients can be harmful, especially among those with cardiac arrest and traumatic brain injury [4, 5]. Several mechanisms have been proposed, including vasoconstrictive effects on the cerebral vasculature, microscopic endothelial dysfunction, and the generation of reactive oxygen species (ROS), after exposure to supraphysiological level of oxygen [9–12].

In the present study, we found that hyperoxia was associated with poor neurological outcomes at 3 months among patients with aSAH. This agreed with a prior study by Yokoyama et al., which demonstrated that early hyperoxia was associated with poor neurological outcomes in SAH patients with Hunt and Kosnik grade I–III [13]. Another study by Jeon et al. similarly found that exposure to hyperoxia was associated with delayed cerebral ischemia (DCI) and poor neurological outcomes [14]. However, such association was not observed in the study performed by Lang et al. [15]. Despite showing a possible association between high time-weighted average of PO_2_ and unadjusted neurological outcome at 3 months, this relationship was diminished after adjustment in multivariate analysis. A possible cause for such contradictory results may be due to the lack of consensus on definition of hyperoxia among the aforementioned studies.

Carbon dioxide is a potent vasodilator of cerebral vasculature. While effectively reducing intracranial pressure (ICP) with hyperventilation, the vasoconstrictive effect of hypocapnia, however, may jeopardize cerebral blood flow (CBF) and exacerbate cerebral ischemia [16–18]. The effects of hypocapnia have been extensively studied in patients with traumatic brain injuries. Prolonged, prophylactic hyperventilation targeting hypocapnia is no longer recommended in current clinical guidelines but reserved as a rescue measure for severe intracranial hypertension [19]. The potential harmful impacts of hyperventilation and the resulting hypocapnia were again observed in the present study among patients with SAH. In our classification tree model, hypocapnia predicted poor neurological outcomes in patients who were less ill and without concomitant ICH/IVH. Similar finding was reported by Williamson et al. that spontaneous hyperventilation in SAH patients was associated with DCI and poor neurological outcomes [20]. A retrospective study by Yokoyama et al. also reported that both hypercapnia and hypocapnia might be associated with unfavorable neurological outcomes [21].

Although hypercapnia was more commonly observed among the recruited patients with poor neurological outcomes, we were unable to demonstrate an association between hypercapnia and neurological outcomes by logistic regression analysis. We postulated that for cases with hypercapnia, drainage of cerebrospinal fluid (CSF) with an external ventricular device may counteract the potentially harmful effects of raised intracranial pressure. In a phase 1 clinical trial by Westermaier et al. gradual hypercapnia was well tolerated among patients with poor grade SAH without causing an increase in ICP when the drainage device was kept open for continuous drainage [22]. The use of sedatives may also inhibit the sympathetic activity and buffer the increases in CBF associated with hypercapnia and the resultant intracranial hypertension.

Apart from age and APACHE IV scores, WFNS and modified Fisher radiological grading scale, two widely used clinical grading scales in clinical practice were again found to predict poor neurological outcomes in the regression analysis. We also found that patients who underwent endovascular interventions had better outcomes. With the advances in interventional radiological techniques, endovascular interventions have gained widespread acceptance for treatment of aneurysms, especially in elderly patients and those with poor grades SAH at presentation. Clinical guidelines and a recent Cochrane review also suggested potential advantage of endovascular coiling over neurosurgical clipping if the aneurysms are amendable by either method [7, 23]. The location of aneurysms also predicted neurological outcomes in recruited patients, with aneurysms located in the posterior circulation being associated with worse outcomes. Similar findings were also reported by Abla et al. that ruptured aneurysms in posterior circulation was associated with worse 1-year modified Rankin scale [24]. This may be due to the proximity of the aneurysmal bleeding to brainstem and lower cranial nerves, and thus patients were more prone to dependence on prolonged ventilatory support or tube feeding due to bulbar dysfunction.

Classification tree analysis was adopted in our study. It enables us to assess the interactions between predictor variables, an advantage over logistic regression analysis which addresses the predictor variable individually [25]. Yet, classification tree models are vulnerable to “overfitting” with identified variables exerting effect only on a specific group of patients and thus limiting its generalization capability. For instance, hypocapnia was associated with poor neurological outcomes only in patients who were less critically ill and without ICH/IVH.

The present study is not without flaw. First of all, it is a retrospective study performed in a single center, which may introduce potential selection bias. Secondly, blood gas results only reflected levels at a single time point when the blood samples were obtained, rather than continuous, real-time measurement. The actual exposure to O_2_ or CO_2_ burden was therefore unknown. Furthermore, time points and intervals of blood gas measurement were inconsistent across patients. Those who were critically ill may have more frequent blood gas sampling. This may be an additional source of bias. In addition, data on parameters of mechanical ventilation, incidence of pulmonary complications such as neurogenic pulmonary edema and aspiration pneumonitis, and the use of pharmacological agents including sedation and muscle relaxants were not available. These confounders may affect patients' O_2_ or CO_2_ burden and thus biased the presented results. Lastly, the GOS score at 3 months after admission was used as the outcome measure in the present study. A follow-up study from the Intraoperative Hypothermia for Aneurysm Surgery Trial (IHAST) found that neurocognitive improvements can be delayed well beyond 3 months after neurological insult, plateauing between 9 and 15 months [26]. A follow-up study is therefore required to carefully assess the long-term neurological outcomes in SAH patients.

## 4. Conclusions

Our results underlined the potentially harmful impacts of hyperoxia and hypocapnia on neurological outcome in patients with aSAH. Hyperoxia independently predicted poor neurological outcome, while hypocapnia was associated with poor neurological outcomes in patients who were less critically ill and without concomitant ICH/IVH. Further prospective studies are necessary to validate our findings before it can be robustly translated into clinical practice.

## Figures and Tables

**Table 1:** Baseline characteristics of patients recruited.

Parameters	All patients (*N* = 244)	GOS 4-5 (*N* = 122)	GOS 1–3 (*N* = 122)	*P* value
Age (years)	57.7 ± 14.6	52.7 ± 13.9	62.8 ± 13.6	<0.001
Male	89 (36.4)	36 (30.0)	53 (43.4)	0.024
Source of admission				0.113
General ward	38 (15.6)	20 (16.4)	18 (14.8)	
Operation room/recovery room	181 (74.2)	94 (77.0)	87 (71.3)	
Others	25 (10.2)	8 (6.6)	17 (13.9)	
APACHE IV score	59 ± 31	42 ± 22	77 ± 28	<0.001
APACHE IV risk of death	0.25 ± 0.24	0.12 ± 0.14	0.37 ± 0.26	<0.001
Day 1 lowest GCS	8 ± 5	11 ± 4	6 ± 4	<0.001
WFNS grading				<0.001
1	97 (39.8)	77 (63.1)	20 (16.4)	
2	22 (9.0)	11 (9.0)	11 (9.0)	
3	18 (7.4)	11 (9.0)	7 (5.7)	
4	40 (16.4)	12 (9.8)	28 (23.0)	
5	67 (27.5)	11 (9.0)	56 (45.9)	
Modified Fisher radiological grading				<0.001
1	35 (14.3)	31 (25.4)	4 (3.2)	
2	26 (10.7)	17 (13.9)	9 (7.4)	
3	54 (22.1)	43 (35.2)	11 (9.0)	
4	129 (52.9)	31 (25.4)	98 (80.3)	
Location of aneurysm				0.002
Anterior circulation	153 (62.7)	90 (73.8)	63 (51.6)	
Posterior circulation	66 (27.0)	24 (19.7)	42 (34.4)	
Not clear	25 (10.3)	8 (6.6)	17 (13.9)	
Aneurysm maximum size (mm)	5.6 ± 5.8	5.4 ± 3.7	5.7 ± 7.3	0.658
Presence of ICH/IVH	155 (63.5)	48 (39.3)	107 (87.7)	<0.001
Presence of vasospasm	101 (41.4)	45 (36.9)	56 (45.9)	0.153
Mild (>100–130 cm/s)	24 (23.8)	15 (33.3)	9 (16.1)	
Moderate (>130–200 cm/s)	57 (56.4)	24 (53.3)	33 (58.9)	
Severe (>200 cm/s)	20 (19.8)	6 (13.3)	14 (25.0)	
Interventions				
Endovascular intervention	136 (55.7)	87 (71.3)	49 (40.2)	<0.001
Surgical clipping	53 (21.7)	31 (25.4)	22 (18.0)	0.162
External ventricular drainage	131 (53.7)	50 (41.0)	81 (66.4)	<0.001
PO2 within 24 hours of ICU admission (mmHg)				
Maximum	151.2 ± 62.6	141.1 ± 43.3	161.4 ± 76.1	0.011
Minimum	92.7 ± 25.6	94.0 ± 26.8	91.4 ± 24.4	0.437
Average	119.6 ± 34.4	116.3 ± 29.9	122.8 ± 38.3	0.142
Hyperoxia >200 mmHg within 24 hours of ICU admission	39 (16.0)	12 (9.8)	27 (22.1)	0.009
PCO2 within 24 hours of ICU admission (mmHg)				
Maximum	41.9 ± 11.5	39.0 ± 6.3	44.9 ± 14.4	<0.001
Minimum	33.1 ± 6.4	32.4 ± 5.2	33.7 ± 7.5	0.095
Average	37.1 ± 7.2	35.6 ± 4.6	38.7 ± 8.8	0.001
Hypocapnia <30 mmHg within 24 hours of ICU admission	81 (33.2)	39 (32.0)	42 (34.4)	0.683
Hypercapnia >45 mmHg within 24 hours of ICU admission	60 (24.6)	16 (13.1)	44 (36.1)	<0.001
Length of stay (days)				
ICU	7.6 ± 6.2	6.8 ± 5.7	8.5 ± 6.6	0.036
Hospital	21.1 ± 27.5	16.5 ± 9.2	25.6 ± 37.3	0.009
Mortality				
ICU	44 (18.0)	0 (0)	44 (36.1)	<0.001
Hospital	59 (24.2)	0 (0)	59 (48.0)	<0.001
30 days	57 (23.4)	0 (0)	57 (46.7)	<0.001
3 months	62 (25.4)	0 (0)	62 (50.8)	<0.001

Data presented as mean ± standard deviation (SD) and number (percentage). Abbreviations: GOS = Glasgow Outcome Scale; APACHE = Acute Physiology and Chronic Health Evaluation; GCS = Glasgow Coma Scale; WFNS = World Federation of Neurosurgical Society; ICH = intracranial hemorrhage; IVH = intraventricular hemorrhage; ICU = intensive care unit; *N* = number.

**Table 2:** Logistic regression analysis for independent predictors for poor neurological outcome.

Parameters	Odds ratio	95% CI	*P* value
APACHE IV score >50	5.403	2.456–11.886	<0.001
Age >55 years old	4.065	1.857–8.898	<0.001
Hyperoxia (PaO2 >200 mmHg within first 24 hours)	3.788	1.131–12.690	0.031
Aneurysm involving posterior circulation	3.685	1.477–9.193	0.005
Presence of ICH/IVH	3.644	1.602–8.292	0.002
Modified Fisher radiological grading >2	3.539	1.465–8.550	0.005
WFNS grading >3	2.937	1.270–6.794	0.012
Underwent intervention radiological procedures	0.382	0.170–0.859	0.020

Hosmer and Lemeshow test *χ*^2^ = 4.070, df = 8, *P*=0.851, Cox and Snell R square 47.4%, Nagelkerke R squared 63.2%, C statistic 0.915 (95% CI 0.879–0.950). Abbreviations: APACHE = Acute Physiology and Chronic Health Evaluation; ICH = intracranial hemorrhage; IVH = intraventricular hemorrhage; WFNS = World Federation of Neurosurgical Society; df = degrees of freedom.

## Data Availability

The data used to support the findings of this study are available from the corresponding author upon request.
